# The Prevalence of Osteopenia and Osteoporosis Among Malaysian Type 2 Diabetic Patients Using Quantitative Ultrasound Densitometer

**DOI:** 10.2174/1874312901812010050

**Published:** 2018-04-25

**Authors:** Shaymaa Abdalwahed Abdulameer, Mohanad Naji Sahib, Syed Azhar Syed Sulaiman

**Affiliations:** 1 Faculty of Pharmacy, Al-Rafidain University College, Palestine Street, 10052, Baghdad, Iraq; 2 School of Pharmaceutical Sciences, Universiti Sains Malaysia, Minden, 11800, Penang, Malaysia

**Keywords:** Diabetes, Malaysia, Osteopenia, Osteoporosis, Prevalence, Quantitative ultrasound

## Abstract

**Background:**

Type 2 Diabetes Mellitus (T2DM) and osteoporosis are both chronic conditions and the relationship between them is complex.

**Objective:**

The aims of this study were to assess the prevalence of Low Bone Mineral density (LBMD, *i.e*., osteopenia and osteoporosis), as well as, the difference and associations between Quantitative Ultrasound Scan (QUS) parameters with socio-demographic data and clinical related data among T2DM in Penang, Malaysia.

**Method:**

An observational, cross-sectional study with a convenient sample of 450 T2DM patients were recruited from the outpatient diabetes clinic at Hospital Pulau Pinang (HPP) to measure Bone Mineral Density (BMD) at the heel bone using QUS. In addition, a self-reported structured questionnaire about the socio-demographic data and osteoporosis risk factors were collected. Moreover, the study included the retrospective collection of clinical data from patients’ medical records.

**Results:**

The mean value of T-score for normal BMD, osteopenic and osteoporotic patients’ were (-0.41±0.44), (-1.65±0.39) and (-2.76±0.27), respectively. According to QUS measurements, more than three quarters of T2DM patients (82%) were at high risk of abnormal BMD. The results showed that QUS scores were significantly associated with age, gender, menopausal duration, educational level and diabetic related data. Moreover, the QUS parameters and T-scores demonstrated significant negative correlation with age, menopausal duration, diabetic duration and glycaemic control, as well as, a positive correlation with body mass index and waist to hip ratio. The current study revealed that none of the cardiovascular disease risk factors appear to influence the prevalence of low BMD among T2DM Malaysian patients.

**Conclusion:**

The study findings revealed that the assessment of T2DM patients’ bone health and related factor are essential and future educational programs are crucial to improve osteoporosis management.

## 1 INTRODUCTION

Osteoporosis condition is recognised as a “silent global problem”, which is characterised by a reduction in the bone mass and micro-architectural deterioration of bone tissue, leading to impaired skeletal strength with a consequent increase in bone fragility and susceptibility to fracture [1, 2]. However, osteoporosis does not have a dramatic clinical presentation and frequently remain undiagnosed until there is a fracture or a screening test is conducted [3, 4]. Moreover, the growth of the aged population is rapidly increasing with increased lifespans of the population due to medical improvements in the developed as well as the developing world, which contributes to a significant increase in the prevalence of osteoporosis with a consequently increased number of fractures [5, 6]. Thus, osteoporosis imposes a significant and heavy burden on the individual and healthcare system [7]. The osteoporotic condition negatively and significantly impacts morbidity as well as can lead to chronic pain, deformity, impair mobility and increased risk of death [8]. In addition, persons with high cumulative rate of fractures were often disabled with decreased functional capacity and poor quality of life [9, 10], as well as, become dependent on others [11, 12].

Bone Mineral Density (BMD) is an important factor linked to bone health and fracture risk. It is well known that BMD in the United States and European Caucasian groups is higher than that in the Asian populations [13-15]. However, with the rise in Asian population, it is predicted that Asian countries will be progressively increasing in the incidence of fractures and more than half of all fractures around the world will occur in Asia by 2050 [16]. Many studies have indicated that Quantitative Ultrasound Scan (QUS) has emerged as a new and adequate tool that offers an alternative or adjunct to Dual energy X-ray Absorptiometry (DXA) for screening and assessing the peripheral skeleton status. Thus, QUS accurately predicts the relative risk of all fracture risks similar to that from DXA measurements of the hip and spine [17, 18]. Moreover, there were many studies that had shown that the T-score resulting from the QUS method were correlated with the T-scores resulting from the DXA method [19, 20]. Furthermore, QUS parameters reflect BMD, as well as, other mechanical characteristics of the bone, such as elasticity, microarchitecture and strength [21, 22]. In addition, QUS can discriminate between individuals with a low risk and high risk of having abnormal BMD in the clinical setting [23, 24]. According to the recommendations of the International Society for Clinical Densitometry, the calcaneus (heel) is the only ideal validated anatomic site for bone mass screening using QUS method because it is weight-bearing and trabecular rich [25, 26].

On the other hand, Type 2 Diabetes Mellitus (T2DM) and osteoporosis are both chronic conditions; the relationship between them is complex. Clinical data uniformly support that bone formation and bone micro-architectural integrity are altered in diabetic patients [27, 28]. Moreover, glucose metabolism impairment has a number of detrimental effects on bone remodeling in terms of reduced bone mass [29, 30] and an increased risk of fractures [31]. In view of the current high prevalence of T2DM (14.90%) [32] and osteoporosis (24.10%) [33] in Malaysia, there is a great interest in studying the possible prevalence of osteoporotic status (normal BMD, osteopenia and osteoporosis) in T2DM patients. To date, there has been no clinical study exploring the prevalence of osteoporotic conditions in Malaysian T2DM patients. Therefore, the present study aimed to assess the prevalence of Low Bone Mineral Density (LBMD, *i.e*., osteopenia and osteoporosis) in T2DM, as well as, the difference and associations between QUS parameters with socio-demographic data and clinical related data.

## 2 MATERIALS AND METHODS

### 2.1 Study design

An observational, cross-sectional study with a self-reported structured questionnaire about the socio-demographic data and osteoporosis risk factor was used for this study. In addition, the study included the retrospective collection of clinical data from patients’ medical records. The study population consisted of diabetes outpatients attending the Diabetes Outpatient Clinic of Hospital Palua Pinang (HPP), from 1^st^ August 2011 to 30^th^ February 2012. The study population was recruited using a convenient sampling method. The inclusion criteria in the present study were: patients diagnosed with T2DM (depending on the medical record) at least two years before inclusion in the study, age ≥ 18 years old and able to read and write with no speech or hearing problems.

### 2.2 Sample Size

The following Cochran formula was used to calculate the required sample size for this study [34]:

$n=\frac{Z^{2}P(1-P)}{d^{2}}$

Where n = sample size, Z = Z statistic for a level of confidence (the value of *Z* is set at 2.58 with a confidence of about 99%), P = expected prevalence or proportion (the T2DM prevalence in Malaysia is 14.9% according to the third National Health and Morbidity Survey (NHMS III) [35, 32]; therefore, P is equal to 0.149), and d = precision (in proportion of one; if 5%, d = 0.05), because it is appropriate to have a precision of 5% if the prevalence of the disease is estimated to be between 10% and 90%. Using an accepted margin of error of 5% and a 99% confidence interval, the required sample size was calculated to be 338 patients. With the inclusion of an additional 40% (to cover study drop-outs) to minimise erroneous results and increase the study reliability, the target sample size was increased to 474 patients.

### 2.3 Bone Mass Measurements

The skeletal health status was evaluated using Quantitative Ultrasound Scan (QUS) measurements to determine the prevalence of LBMD at the heel bone (calcaneus) and these measurements was carried out by the SONOST 3000 clinical bone densitometer (OsteoSys Co., Ltd, Seoul, Korea). Quality assurance tests were run on a daily basis according to the manufacturer’s protocol with the standard phantom before each data collection session to ensure the stability of QUS measurements. The QUS measurements were obtained from the right calcaneus for all patients in a room temperature controlled environment, as suggested by the manufacturer, and the measurements were conducted by the same investigator throughout the studies.

The QUS measures two basic parameters, Broadband Ultrasound Attenuation (BUA) and the Speed Of Sound (SOS), in addition to the T-score, which is comparable to that obtained by DXA. The BUA, expressed in decibels/megahertz (dB/MHz), is concerned with the structural features of bones and is a measurement of different attenuations of the ultrasound waves transmitted through the calcaneus [36, 37]. In contrast, SOS, expressed in meters/second (m/s), is concerned with bone strength, fragility and elasticity, which translates the time necessary for ultrasound to travel through the calcaneus [38, 26, 39].

Moreover, Stiffness Index (SI) and estimate Bone Mineral Density (eBMD) parameters were generated from both BUA and SOS. The SI and eBMD, expressed in percent (%) and g/cm^2^, respectively, are variables derived from a linear combination of BUA and SOS and calculated according to the following equations [40, 41]:

SI=((0.67×BUA)+(0.28×SOS)−420)

eBMD = (0.0025926 × (BUA + SOS)−3.687)

Some studies have shown that SI has the best correlation with BMD (measured by DXA) and fracture risk [38, 42, 43]. The QUS-score measurements were categorised into three groups (normal BMD, osteopenia and osteoporosis) according to the World Health Organization (WHO) criteria which have been standardised by the manufacturer for Asian population. Osteoporosis, osteopenia and normal conditions are identified as (T-score ≤ -2.5 SD), (T-score between -1.0 and -2.5) and (T-score > -1.0) below the healthy young adult reference mean, respectively [44]. In addition, osteoporotic conditions of the T2DM patients were stratified into two groups: a normal group (low risk for abnormal BMD with T-score > -1) and an osteoporotic condition group (high risk for abnormal BMD with T-score ≤ -1 (i.e. osteopenia and osteoporosis) [45].

### 2.4 Ethical Considerations

Before the initiation of this study, all aspects of the study protocol were approved by the Clinical Research Centre (CRC) of HPP and the Medical Research Ethics Committee (MREC) of the Ministry of Health, Malaysia (NMRR-11-28-8209). All subjects were provided with a written informed consent form prior to participation in this study. All personal information collected was considered confidential.

### 2.5 Statistical Analysis

Data obtained from the interviews and medical records were checked to ensure completeness. All statistical analyses were performed using Predictive Analytics Software (PASW) version 19.0 and the level of statistical significance was set less than 0.05 (*P*<0.05). Descriptive statistics representing mean ±standard deviations (M±SD), percentages (%), ranges, and 95% Confidence Intervals (CI) were used to describe demographic and disease characteristics of the patients. Percentages and frequencies were used for categorical variables and the associations between categorical variables were examined using chi square (χ^2^) test. Continuous normally distributed variables were compared using the One Way Analysis Of Variance (ANOVA) test, Analysis Of Covariance (ANCOVA) and independent student t-test when appropriate. Post hoc analysis using Hochberg’s GT2 was conducted when necessary. The Hochberg’s GT2 test was used because of the unequal sample size among the groups [46]. However, the Kruskal Wallis test was used for continuous non-normal distribution data. Spearman and Pearson correlation were used to measure the correlation between continuous variables when appropriate.

## 3 RESULTS

### 3.1 Overall Response Rate

A total of 500 patients were recruited from the outpatient diabetes clinic. Out of the 500 patients approached, 50 patients were excluded due to a lack of some clinical data (n=31) or incomplete responses from the patients (n=19). The final convenience sample of 450 patients with T2DM was included in the analysis of this study, which was considered to be acceptable with a margin of error (±5%) and 99% confidence interval [47].

### 3.2 Bone Health Status and Prevalence of Osteoporotic Conditions

All T2DM patients were screened for BMD using QUS measurement. The mean value of T-score for the total sample (N=450) was (-1.67±0.83) ((median: -1.65) (range: -3.4 to 1.2) (CI 95%: -1.75 to -1.59)). The mean value of T-score for normal BMD, osteopenic and osteoporotic patients’ were (-0.41±0.44), (-1.65±0.39) and (-2.76±0.27), respectively. In addition, the mean values of the QUS parameters as following: BUA, SOS, SI and eBMD were 67.09 ±15.40 dB/MHz, 1514.37±14.26 m/s, 48.98±13.53% and 0.41 ±0.07 gm/cm^2^, respectively. According to QUS measurements, the prevalence of normal BMD was 18% (n=81), while the prevalence of osteopenia and osteoporosis were considered as 59.8% (n=269) and 22.2% (n=100), respectively.

### 3.3 Bone Health Status and Socio-Demographic Data

The QUS measurements identified 369 patients (82%) out of 450 with high risk of abnormal BMD. The socio-demographic and osteoporotic conditions are presented in Table **1**. The results showed a higher proportion of males with a low risk of abnormal BMD (67.90%) and a higher proportion of females with a high risk of abnormal BMD (52.30%) (*P*<0.05). Moreover, there were significant differences in QUS-score (normal BMD, osteopenia and osteoporosis) with age, menopausal duration, anthropometric indicators (BMI, WHR) and educational level (*P*<0.05).

There was a significant difference in QUS-score with age (*P*<0.05). Post hoc analysis (Hochberg’s GT2) showed that the osteoporotic and normal groups were significantly different with age (*P*< 0.05), while there were insignificant differences between the osteopenia group and both osteoporosis and normal groups (*P*>0.05) regarding mean age. However, the results showed that the mean age of the osteoporosis group (64.19 ± 8.62 years) was higher than that of both the osteopenic (62.68 ± 9.19 years) and normal (60.78 ± 9.88 years) groups. In addition, there was a significant difference in QUS-score when stratified according to menopausal duration (*P*<0.05). The mean menopausal duration of the osteoporosis group (16.93±7.42) was found to be higher than that of both the osteopenia (15.37±7.80) and normal (10.32±8.59) groups.

In short, the anthropometric measurement values for the osteopenia and osteoporosis groups were significantly lower than for the normal group (*P*<0.05). In addition, higher proportion of osteopenia (68.40%) and normal BMD (48.10%) among those with an educational level less than 12 years than in those with more than 12 years, respectively (*P*<0.05); however, there was an insignificant difference in the proportion between educational levels with osteoporosis group (*P*>0.05). Finally, the results showed insignificant associations between the osteoporotic status (normal BMD, osteopenia and osteoporosis) with race, income, menopausal status, living place, employment status, family history of osteoporosis, family history of fracture, alcohol and smoking habits (*P*>0.05), as shown in Table **1**.

Furthermore, the results showed significant negative correlations between T-score and QUS parameters with age in years, menopause duration (*P*<0.05) (Table **2**). On the other hand, positive correlations between T-score and QUS parameters with BMI and WHR (*P*<0.05) were found.

### 3.4 Quantitative Ultrasound Parameters Measurements’ with Age and Gender

The QUS parameters of the calcaneus stratified by age decade and gender are presented in Table **3**. The present study showed that the parameters of QUS (BUA, SOS, eBMD, SI) gradually decreased in both sexes with increasing age. Men presented higher values for all parameters in comparison to women. In addition, higher values for all parameters, in both sexes, were detected in younger age groups (less than 45 years). There were significant effects of age on the levels of SOS, BUA, SI and eBMD after controlling for the effects of gender and race (*P*<0.05). In addition, the covariate race was insignificantly related to all parameters (*P*>0.05). Furthermore, the covariate gender was significantly related to all parameters except SOS (*P*>0.05).

### 3.5 Bone Health Status with Clinical Related Data

In this study, significant associations between QUS-score and glycaemic control (HbA1c) and diabetes duration were found (*P*<0.05), respectively. The results showed a higher proportion of osteoporosis (83%) in poor glycaemic control (HbA1c≥6.5) and higher proportion of normal BMD (34.60%) among good glycaemic control patients (HbA1c<6.5), respectively (P<0.05); however, there was an insignificant difference in proportion between glycaemic control with osteopenia group (P>0.05). Moreover, the results showed significantly a higher proportion of osteopenia (34.20%) and osteoporosis (43%) in the group with a diabetes duration of more than 10 years and a higher proportion of normal BMD (81.50%) in the group with a diabetes duration of less than 10 years (P<0.05). In addition, there were insignificant association between QUS-score and the other diabetes-related variables (*P*>0.05) as shown in Table **4**. Furthermore, insignificant associations between QUS-score with lipid and blood pressure profiles variables (data not shown) (*P*>0.05).

Moreover, significant negative correlations were found between T-score and QUS parameters with glycaemic control (HbA1c) and duration of diabetes, as well as Systolic Blood Pressure (SBP) (*P*<0.05). In addition, insignificant correlations were found between T-score and QUS parameters with both lipid and blood pressure profiles (*P*>0.05), as shown in Table **5**.

## 4 DISCUSSION

Diabetes mellitus and osteoporosis are two clinical syndromes with a great impact on public health that affect a large proportion of people around the world. The clinical relevance of osteoporosis related to T2DM is less acknowledged and, to date, no clear findings have been reached due to the inconsistent findings among researchers; they have reported lower, equal and greater bone mass in T2DM [48]. To the best of the researchers’ knowledge, this was the first extensive field study using calcaneal QUS to determine and identify the prevalence of osteoporosis and osteopenia in Malaysian diabetic type 2 patients in Penang state.

### 4.1 Prevalence of Osteoporotic Conditions

This study showed that T2DM patients had a high prevalence of osteopenia (59.80%) and osteoporosis (22.20%). Comparable results were found in other studies using QUS [49] and DXA [50]. However, the prevalence of osteoporosis in the present study was lower than other studies of Asian populations [51, 52] and other western country [53]. On the other hand, the overall prevalence of osteoporosis in this study was higher than those reported in several other studies in different countries, using either DXA [54, 55] or QUS [56, 10]. Therefore, it appears that osteoporosis in Malaysian T2DM patients is underdiagnosed and overlooked until now.

### 4.2 Quantitative Ultrasound Parameters Measurements

The QUS parameters demonstrated significant inverse correlations with age. Moreover, the results showed an obvious decrease in all QUS parameters (BUA, SOS, eBMD, SI) with age and the average values for men were higher than those for women. Similar findings were seen by other researchers using the QUS in the general population [57, 58]. Moreover, similar evidence from previous cross-sectional studies in European [59, 60] and Asian populations [61, 21] using the QUS measurement have reported that QUS parameters start to decline from the age of 40–45 years and continue to decrease rapidly with age.

The peak values for the QUS parameters in the present study occurred in the age group younger than 45 years, which are consistent with other studies [62, 63]. In agreement with previous reports, the decline of BMD in Asian men is slower than in women, especially after the age of 50 years [64, 65]. Moreover, osteoporosis was more common among women compared to men using calcaneal QUS measurements [66, 10]. Furthermore, the decline in women within this study was more prominent after the age of 55 years. The differences in age at peak bone mass and the beginning of bone mass reduction could be partly explained by the characteristics of bone (skeletal sites), as well as, the measurement methods used [67].

The prevalence of osteoporosis and osteopenia was higher in females than males. It was generally assumed that osteoporosis is less common in men than women. This is because men has shown greater BMD at all areal sites and the average percent loss in BMD for men was much lower than the loss for women at all sites [68, 69]. Other studies showed higher or no difference in BMD in T2DM men when compared with healthy controls [70-72]. However, many studies revealed lower BMD among men T2DM patients when compared to healthy controls [73, 74], as well as, higher prevalence of osteoporosis in diabetic men after adjustment for age and BMI [75]. It was estimated that each year, there were approximately 1% BMD losses in men with diabetes [76] and the risk of fall related fracture was increased due to the decline in muscular strength and neuromuscular function [74]. Thus, the problem of osteoporosis in men has been overlooked.

### 4.3 Socio-Demographic Data and Bone Health Status

The QUS parameters demonstrated significant inverse correlations with menopausal duration and positive correlations with BMI and WHR. These results are largely consistent with those found in Caucasian and Asian populations using the QUS method [77, 78]. Similar findings were reported in diabetes patients in a Persian study which showed that T-score using DXA was inversely related to the duration of menopause [79]. Another study showed that the duration of menopause, as well as, the age and duration of diabetes, were amongst risk factors for decreasing BMD in diabetic patients [80]. It is obviously known that osteoporosis is a problem related to age and hormonal changes in women, suggesting a hormonal influence on BMD [81]. Moreover, similar findings were reported in postmenopausal women with T2DM using QUS [82]. This might be due to the fact that there is an increase in bone resorption relative to formation as a consequence of aging, which is an important cause of osteoporosis in the elderly [12].

The high prevalence of osteoporosis in Malaysian postmenopausal women is probably related to their short, small skeletal frame and the mainly sedentary lifestyles [83, 33]. However, the osteoporosis prevalence in postmenopausal women with T2DM in the present study was lower than those reported in other studies of postmenopausal T2DM patients using DXA [84, 85] or QUS for non-diabetic postmenopausal women [86, 87]. On the other hand, the current study results showed a higher prevalence of osteoporosis than another study using DXA in T2DM Iranian postmenopausal women [88], or using QUS among Vietnamese, Korean and Indian postmenopausal women [89-91]. The reason behind the differences in the prevalence of low bone mass reported in different countries can be due to the differences in the individual’s lifestyles variables, the method used for measurements and the study design. Additionally, in this study, QUS parameters and the T-score positively correlated with BMI and WHR. Similar findings were reported in other studies, as QUS parameters were inversely correlated with age and positively correlated with weight and BMI; however, no correlation was found with height [92, 93].

### 4.4 Clinical Related Data and Bone Health Status

In the present study, the result revealed that more than three quarters of T2DM patients presenting with osteoporosis (83%) had poor glycaemic control and that an increased diabetic duration of more than 10 years led to a high prevalence of osteopenia and osteoporosis. Moreover, the T-scores and QUS parameters demonstrated significant inverse correlations with glycaemic control (HbA1c) and diabetes duration. Therefore, it is important to maintain a good glycaemic control, as this will lead to slower bone loss in T2DM patients.

Several studies confirm the finding of the current study that glycaemic control is considered to be a confounding factor that affects BMD. In these studies, BMD was more severely decreased in the poorly glycaemic controlled T2DM patients; thus, the bone health status could be improved by maintaining good glycaemic control [94, 95]. On the other hand, other studies have shown that higher BMD was associated with poor glyceamic control in T2DM patients [96, 97] and could be explained by their hyperinsulinaemia and insulin resistance [98, 99]. Several studies indicated that insulin has shown a potential anabolic effect on skeleton tissue [100, 101], as well as, it is responsible for increased BMD in T2DM [102]. Thus, elevated BMD in T2DM, at least in the early phase, hallmarked by increased insulin level [103], then later when the pancreases cannot be able to secrete enough insulin to overcome the increased insulin resistance that results in periods of hyperinsulinemia [104].

On the other hand, some studies have shown that there was no relationship between T-score and HbA1c [105, 106], which was in accordance with the results from a meta-analysis performed in diabetic patients [107]. Thus, it is possible that the effects of hyperglycaemia and poor glycaemic control are not associated with increased bone resorption and bone turnover in T2DM and that BMD is not altered in T2DM [108]. Meanwhile, fractures are more commonly reported in T2DM patients with chronic diabetic complications [109, 110]; therefore, the HbA1c in T2DM patients is still a significance factor, although some previous researchers have shown no direct association between that HbA1c and BMD.

In this study, the results showed that increased diabetic duration (more than 10 years) leads to a high prevalence of osteopenia and osteoporosis. In addition, there was a negative correlation between T-score and QUS parameters with diabetes duration. These result consistent with the previous reported research [106]. Similar findings suggested that age and the duration of diabetes were additional risk factors for developing osteoporotic conditions, as BMD was inversely correlated with age and the duration of diabetes [111, 112]. In contrast, other study did not find any significant relationship between the duration of diabetes and BMD [105]. This inconsistency may be due to the non-randomised control study with small sample sizes, different durations of diabetes and different sites of measurements.

This study only targeted outpatients with Type 2 Diabetes Mellitus in HPP, who may not represent all Malaysian diabetic patients. However, the sample size was large enough to represent all diabetic patients managed in the outpatient clinic of HPP. The convenient sampling and cross-sectional design study further limits the generalisation of the findings to the entire Malaysian population. Moreover, unequal numbers of ethnic, age, and gender groups may impact the final results if compared with a randomised control study or longitudinal study.

## CONCLUSION

This study found that diabetic patients had a high risk of abnormal BMD. As there is evidence that osteoporosis is a preventable disease, the screening, identification and prevention of potential risk factors for osteoporosis in T2DM patients is crucial. It would be appropriate to offer an educational program to individuals about healthy lifestyles that could potentially prevent or control osteoporosis, like engaging in physical activity, maintaining a healthy body mass, minimising the use of tobacco and alcohol, and ingesting appropriate nutrition (adequate dietary intake of calcium and vitamin D). This action may contribute to healthy bones and play a role in the practical prevention of osteoporosis in Asian population.

## Figures and Tables

**Table 1:** The main patient characteristics regarding the prevalence of osteoporosis and osteopenia among type 2 diabetic patients (N=450).

**Variable**	**Total Sample (M±SD) or** **N (%)**	**QUS score, N (%)**			***P* value**
		**Normal BMD**	**LBMD**		
		**Normal**	**Osteopenia**	**Osteoporosis**	
		**81(18.0%)**	**269 (59.8%)**	**100(22.2%)**	
**Age (years)**	62.67±9.24	60.78±9.88	62.68±9.19	64.19±8.62	0.047 ^a*^
**Menopausal age (years) (N= 194)**	48.81±1.83	49.50±2.19	48.88±1.71	48.30±1.88	0.019 ^b*^
**Menopausal duration (years) (N= 194)**	15.16±7.98	10.32±8.59	15.37±7.80	16.93±7.42	0.003 ^b*^
**BMI, kg/m^2^**	26.36±4.39	29.33**±**4.99	25.64**±**3.83	25.88**±**4.34	0.000 ^b*^
**Waist to hip ratio**	0.911±0.06	0.93±0.063	0.91±0.06	0.91±0.06	0.006 ^a*^
**Gender**					0.004 ^c*^
Male	231(51.3)	55(23.8)	126(54.5)	50(21.6)	
Female	219(48.7)	26(11.9)	143(65.3)	50(22.8)	
**Race**					0.572 ^c^
Malay	127 (28.2)	27(21.3)	73(57.5)	27(21.3)	
Chinese	204 (45.3)	30(14.7)	128(62.7)	46(22.5)	
Indian	119 (26.4)	24(20.2)	68(57.1)	27(22.7)	
**Educational levels**					0.015 ^c*^
<12 years	285 (63.3)	42(14.7)	184(64.6)	59(20.7)	
≥ 12 years	165 (36.7)	39(23.6)	85(51.5)	41(24.8)	
**Monthly income**					0.868 ^c^
Less than RM 2000	330 (73.3)	61(18.5)	195(59.1)	74(22.4)	
More than RM 2000	120(26.7)	20(16.7)	74(61.7)	26(21.7)	
**Menopausal status (N=219)**					0.602 ^c^
Premenopausal	25 (11.4)	4(16.0)	17(68.0)	4(16.0)	
Postmenopausal	194 (88.6)	22(11.3)	126(64.9)	46(23.7)	
**Employment status**					0.527 ^c^
Working	192 (42.7)	30(15.6)	118(61.5)	44(22.9)	
Not working	258 (57.3)	51(19.8)	151(58.5)	56(21.7)	
**Living place**					0.074 ^c^
Rural	90 (20)	10(11.1)	54(60.0)	26(28.9)	
Urban	360 (80)	71(19.7)	215(59.7)	74(20.6)	
**Family history of osteoporosis**					0.451 ^c^
No	392 (87.1)	73(18.6)	230(58.7)	89(22.7)	
Yes	58 (12.9)	8(13.8)	39(67.2)	11(19.0)	
**Family history of Fracture**					0.258 ^c^
No	359 (79.8)	70(19.5)	211(58.8)	78(21.7)	
Yes	91 (20.2)	11(12.1)	58(63.7)	22(24.2)	
**Smoking habit**					0.853 ^c^
Not smoking	318 (70.7)	59(18.6)	190(59.7)	69(21.7)	
Smoking	132 (29.3)	22(16.7)	79(59.8)	31(23.5)	
**Alcohol habit**					0.122 ^c^
Non alcoholic	356 (79.1)	70(19.7)	212(59.6)	74(20.8)	
Alcoholic	94 (20.9)	11(11.7)	57(60.6)	26(27.7)	

^a^ Continuous data are presented as mean ±standard deviation (M±SD) and *P* values were derived from one-way analysis of variance (for continuous variables normal distributed), ^b^ Continuous data are presented as mean ±standard deviation (M±SD) and *P* values were derived from Kruskal Wallis test (for continuous variables not normal distributed), ^c^ Categorical variables, expressed as frequency (percentage, %) of sample and *P* values were derived from the Chi-square test, *p < 0.05. QUS, quantitative ultrasound; BMD, bone mineral density; LBMD, low bone mineral density; RM, Ringgit Malaysia.

**Table 2:** Correlation between T-score and quantitative ultrasound scan parameter values with socio-demographic data (N=450).

**Variables**	**T-score**	**BUA**	**SOS**	**SI**	**eBMD**
Age (years), *r*	-0.173**	-0.170**	-0.156**	-0.176**	-0.175**
Menopausal age (years), *r_s_*	0.090	0.135	0.043	0.124	0.108
Menopausal duration (years), *r_s_*	-0.207**	-0.194**	-0.192**	-0.212**	-0.208**
BMI (kg/m^2^), *r_s_*	0.188**	0.200**	0.159**	0.201**	0.196**
Waist to hip ratio, *r*	0.099*	0.139**	0.069	0.126**	0.113*

BUA, broadband ultrasound attenuation; SOS, speed of the sound; SI, stiffness index; eBMD, estimated bone mineral density; BMI, body mass index. *P<0.05, **P<0.01

**Table 3:** The quantitative ultrasound scan parameters of the calcaneus stratified by age groups and gender (N=450).

**Variables**	**Gender**							
	**Female**				**Male**			
**Age group**	**BUA (dB/MHz)**	**SOS** **(m/s)**	**SI %**	**eBMD** **(g/cm^2^)**	**BUA** **(dB/MHz)**	**SOS** **(m/s)**	**SI %**	**eBMD** **(g/cm^2^)**
>45 years	78.96±15.50	1516.84±15.57	57.62±14.04	0.45±0.08	77.35±21.81	1520.10±20.83	57.45±19.78	0.45±0.11
45-54 years	70.92±14.69	1519.98±14.82	53.11±13.34	0.43±0.07	70.68±14.81	1514.99±14.69	51.56±13.09	0.42±0.07
55-64 years	62.13±14.05	1511.87±12.79	44.95±12.37	0.39±0.07	70.18±15.92	1516.58±14.96	51.66±14.03	0.43±0.07
≥65 years	62.14±12.48	1510.94±12.74	44.69±10.92	0.39±0.06	68.32±16.53	1514.21±14.44	49.76±14.38	0.41±0.08
Total	64.52±14.22	1513.35±13.69	46.96±12.59	0.40±0.07	69.54±16.11	1515.35±14.74	50.89±14.12	0.42±0.07

BUA, broadband ultrasound attenuation; SOS, speed of sound; SI, stiffness index; eBMD, estimated bone mineral density. Values are shown as mean± standard deviation (M± SD).

**Table 4:** Relationships between quantitative ultrasound scan score and diabetes-related variables (N= 450).

**Variable**	**Total Sample (N=450)** **N (%)**	**QUS Score, N (%)**			***P*** value
		**Normal BMD**	**LBMD**		
		**Normal**	**Osteopenia**	**Osteoporosis**	
		**81(18.0%)**	**269(59.8%)**	**100(22.2%)**	
**Diabetes Duration (years)**					0.002*
Less than 10 years	300(66.7%)	66(22.0)	177(59.0)	57(19.0)	
More than 10 years	150(33.3)	15(10.0)	92(61.3)	43(28.7)	
**Diabetic complication (DC)**					0.186
Positive (with DC)	330(73.3)	62(18.8)	189(57.3)	79(23.9)	
Negative (without DC)	120(26.7)	19(15.8)	80(66.7)	21(17.5)	
**Co-morbidities**					0.553
Positive (with Co-morbidities)	426(94.7)	75(17.6)	257(60.3)	94(22.1)	
Negative (without Co-morbidities)	24(5.3)	6(25.0)	12(50.0)	6(25.0)	
**Insulin use**					0.369
With insulin	67(14.9)	16(23.9)	36(53.7)	15(22.4)	
Without insulin	383(85.1)	65(17.0)	233(60.8)	85(22.2)	
**Glycaemic control**					0.020 *
Good HbA1c(< 6.5)	107(23.8)	28(26.2)	62(57.9)	17(15.9)	
Poor HbA1c (≥ 6.5)	343(76.2)	53(15.5)	207(60.3)	83(24.2)	

Chi-square test, *P < 0.05.

QUS, quantitative ultrasound; BMD, bone mineral density; LBMD, low bone mineral density.

**Table 5:** Correlation between T-score and quantitative ultrasound scan parameter value with clinical data (N=450).

**Variables**	**T-score**	**BUA**	**SOS**	**SI**	**eBMD**
Diabetes duration, *r_s_*	- 0.114*	-0.082	-0.122**	-0.097*	-0.108*
Glycaemic control, *r_s_*	- 0.190**	-0.152**	-0.193**	-0.169**	-0.185**
TC, *r*	0.083	0.070	0.088	0.079	0.084
HDLC, *r_s_*	-0.020	-0.045	0.002	-0.034	-0.025
LDLC, *r*	-0.001	-0.004	0.002	-0.003	-0.001
TG, *r_s_*	-0.023	-0.006	-0.035	-0.010	-0.016
SBP, *r_s_*	-0.087	-0.095*	-0.073	-0.099*	-0.092*
DBP, *r_s_*	0.041	-0.003	0.062	0.015	0.031

*r*, Pearson correlation; *r_s_*, Spearman correlation; BUA, broadband ultrasound attenuation; SOS, speed of the sound; SI, stiffness index; eBMD, estimated bone mineral density; BMI, body mass index; TC, total cholesterol; HDLC, high density lipoprotein cholesterol; LDLC, low density lipoprotein cholesterol, TG, triglyceride, SBP, systolic blood pressure; DBP, diastolic blood pressure; *P<0.05, **P<0.01.
